# Supporting Tourism by Assessing the Predictors of COVID-19 Vaccination for Travel Reasons

**DOI:** 10.3390/ijerph19020918

**Published:** 2022-01-14

**Authors:** Cezar Morar, Alexandru Tiba, Tamara Jovanovic, Aleksandar Valjarević, Matthias Ripp, Miroslav D. Vujičić, Uglješa Stankov, Biljana Basarin, Rade Ratković, Maria Popović, Gyula Nagy, Lajos Boros, Tin Lukić

**Affiliations:** 1 Department of Geography, Tourism and Territorial Planning, University of Oradea, 410087 Oradea, Romania; cmorar@uoradea.ro; 2 Department of Psychology, University of Oradea, 410087 Oradea, Romania; 3 Department of Geography, Tourism and Hotel Management, Faculty of Sciences, University of Novi Sad, 21000 Novi Sad, Serbia; tamara.jovanovic@dgt.uns.ac.rs (T.J.); vujicicm@gmail.com (M.D.V.); ugljesa.stankov@dgt.uns.ac.rs (U.S.); biljana.basarin@gmail.com (B.B.); lukic021@gmail.com (T.L.); 4 Faculty of Geography, University of Belgrade, 11000 Belgrade, Serbia; aleksandar.valjarevic@gef.bg.ac.rs; 5 Organisation of World Heritage Cities, 93047 Regensburg, Germany; matthiasripp@posteo.de; 6 Faculty for Business in Tourism, 85310 Budva, Montenegro; dekan@fbt-budva.me (R.R.); maria.popovic@fbt-budva.me (M.P.); 7 Department of Economic and Social Geography, University of Szeged, 6720 Szeged, Hungary; geo.nagy.gyula@gmail.com (G.N.); borosl@geo.u-szeged.hu (L.B.)

**Keywords:** COVID-19, SARS-CoV-2, perceived risk, safety of vaccines, conspiracy beliefs, intention to vaccinate, travel, tourism

## Abstract

The persistence of the SARS-CoV-2 virus imposed vaccination passports for traveling in most countries. We investigated psychological factors that predict the intention to vaccinate for travel. In a cross-sectional study, we examined how demographic variables, vaccination status, perceived risk of infection and severity of disease contracted at travel destination, safety and effectiveness of vaccines against contracting COVID-19 during travel, and conspiracy beliefs are related to intention to vaccinate for travel. Further analyses involved differences between vaccinated and unvaccinated individuals in a Romanian sample regarding conspiracy beliefs, attitudes about vaccines, and self-efficacy of controlling COVID-19 infection. Results showed that the intention to vaccinate for travel reasons is best predicted by vaccination status and perceptions of safety and efficacy of vaccines against COVID-19. Thus, vaccinated individuals believing that vaccines are safe and effective most probably will take another vaccine booster if it will allow them to travel. Positive relationships of the intention to vaccinate for travel reasons were found with age, vaccination status, conspiracy beliefs, perceptions of safety and effectiveness of vaccines, intention to travel, and a more cautious approach to travel. No significant relationships were found between perceptions of risk for self or for transmitting the disease to others, severity of disease, and the intention to vaccinate for travel. We also found significant differences between vaccinated and unvaccinated participants, as unvaccinated participants showed higher levels of conspiracy beliefs and less trust in the safety and efficacy of vaccines. We conclude that campaigns focused on promoting information on the safety and efficacy of vaccines is the most important direction for promoting vaccination in young travelers.

## 1. Introduction

The SARS-CoV-2 pandemic generated unprecedented effects caused by a virus in the modern era, leading to the infection of 261,477,973 people by the end of November 2021, with over 5,215,142 deaths worldwide [1]. In the same time, because of the COVID-19 pandemic, the global economy registered the worst recession since World War II (the global GDP loss was 4.5 % in 2020) [2]. All hopes for salvation against this deadly virus were based on the development of an effective vaccine. In this context, many vaccines were developed, some of them being approved in Europe and North America. Although the vaccines proved to be highly effective against the deadly forms of infection with SARS-CoV-2, the rejection and hesitancy of vaccination by the population further spurs the problem.

In Europe and North America, the vaccination process reached higher rates, resulting in the majority of the population being vaccinated, as the majority (up to 95.2% of individuals) were willing to vaccinate [3], while only a small part of the population refused vaccination (e.g., the share of people vaccinated fully against COVID-19 on 27 November 2021, was 87.78% in Portugal, 80.40% in Spain, and 76.10% in Canada [4]). However, at the continental level, regional level imbalances exist, e.g., the south-eastern regions of Europe undertook a different course (e.g., the share of people vaccinated against COVID-19, on 27 November 2021, was 37.6% in Romania and 25.3% in Bulgaria) [5]. In these countries, a large part of the population remained unvaccinated, mostly related to vaccination hesitancy and refusal. As stated by World Health Organization (WHO) [6], vaccine refusal is one of major threats to world health. This situation resulted in serious consequences of COVID-19 infection in unvaccinated versus vaccinated countries. In this perspective (between 1–31 October 2021), in Romania, there were recorded 10917 deaths, and in Bulgaria 3030 deaths, compared with the much lower values of the deaths because of COVID-19 in the countries with a higher vaccination rate (e.g., 179 deaths in Portugal, 912 deaths in Spain, or 1049 deaths in Canada in the same analyzed period (1–31 October 2021) [4]. Most countries imposed travel restrictions based on the status of vaccination. Given this, vaccination for travel reasons is an important behavior. Furthermore, the possibility that SARS-CoV-2 virus will be present in the future in some regions imposes the necessity of vaccination for travel reasons to individuals traveling from or to regions with higher rates of COVID-19 infections. Assessing the predictors of vaccination for travel reasons may help our understanding of vaccination behavior [7,8,9] and the involved risks potentially harmful to human health and to the physical built environment [10,11,12,13,14].

Research on COVID-19 vaccination followed two directions. The first line comprised research based on surveys exploring the reasons underlying vaccination intention or hesitancy [15,16]. Although different professional groups reported different reasons for vaccination [17], most often participants endorsed the protection of family members [15,16,18] and self-protection [15,16] against COVID-19 infection among the main reasons people vaccinate. Perceiving COVID-19 infection as less severe [17,19,20,21] and the reduced safety and effectiveness of vaccines were among the main reasons for refusal [15,16,19,22,23]. National surveys in Romania populations revealed similar reasons for getting vaccinated. For instance, Romanian respondents endorsed protecting themselves from getting sick (53%), protecting others (47%), desire for socializing, traveling, and going on vacation (42%), desire to feel relaxed (33%), and traveling abroad (13%) as several of the main reasons for getting the vaccine [24].

The second line of research investigated psychological factors associated with vaccine hesitancy and intention to vaccinate. Various psychological factors are involved in psychological vulnerabilities to general health-related risk behaviors [25,26,27,28,29,30]. Furthermore, psychological factors have been revealed to play significant roles affecting vulnerabilities to particular health-related risk behaviors such as vaccination hesitancy [23]. One factor expected to interfere with intention to vaccinate was conspiracy beliefs. Consistent with this hypothesis, research showed that individuals with high levels of conspiracy beliefs [20,31,32] regarding the vaccination seem to be less willing to vaccinate. Another factor is the perceived risk and severity of disease [19]. Previous studies suggest that when individuals perceive a higher probability and severity of disease, they are more willing to vaccinate [19,22,23]. The same relationship has been observed with COVID-19 vaccines: the higher the risk and the perceived severity of COVID-19, the higher the willingness to vaccinate [33,34,35]. Safety and efficacy of vaccines was another factor that impacts vaccination intention. When vaccines are perceived as safe and effective, we expect that individuals will be more willing and less hesitant to vaccinate. Recent research has confirmed this expectation with COVID-19 vaccines, showing a positive relationship between safety and effectiveness of COVID-19 vaccines and willingness to vaccinate [23,36]. Nonetheless, previous experience with vaccines such as previous refusal of vaccination seems to be a salient factor affecting willingness to vaccinate [23,37,38].

Previous research on non-COVID-19 vaccine-preventable diseases showed that vaccination is predicted by perceptions of risk and severity of disease [39,40,41,42,43], prosocial motives [44,45], and individual characteristics, such as socioeconomic status, age, and gender [46,47,48]. These results are in line with studies investigating COVID-19 factors affecting vaccination. Research that investigated factors involved in the acceptance of vaccination to prevent vaccine-preventable disease (malaria, yellow fever, Japanese encephalitis and so on) showed that intention or hesitancy to vaccinate for traveling in endemic countries is influenced by similar factors. For instance, vaccination against malaria or yellow fever before travel is predicted by perceived risk and severity of disease and perceived safety and effectiveness of vaccines [49,50,51,52,53]. Furthermore, distinct types of travelers (travelers visiting friends and relatives) are less likely to vaccinate than other types of travelers (tourists) [54,55].

Recently, in an endemic evolution of COVID-10, COVID-19 vaccination certificates for travel [56] have been proposed as a measure to restore tourism. Because of the time-limited protection effect of COVID-19 vaccines, before-travel vaccinations boosters may be required. Several countries have already required recent vaccination boosters as a travel prerequisite [57]. Thus, knowledge about factors that affect vaccination for travel reasons is necessary to understand people’s reactions to vaccination for travel. Yet, an important gap in the literature is that little knowledge exists regarding factors that influence the intention to vaccinate against SARS-CoV-2 for travel reasons. Knowing processes that affect vaccination for travel may inform adequate measures for a travel medicine approach to COVID-19 vaccination. Furthermore, studying psychological mechanisms involved in disease-related behaviors (vaccination hesitancy or intention) and psychological dysfunctions related to travel point to the importance of travel clinical psychology in the COVID-19 pandemic.

The study will be built on previous knowledge regarding vaccination for travel. Vaccination is already required for traveling to specific areas, for individual protection from various serious diseases on one hand, and for decreasing the risk of spreading viruses to non-endemic areas on the other hand [58]. Travel-related vaccination deals with the routine vaccines before travelling (part of national childhood immunization programs), with the vaccines recommended for certain destinations (to provide protection against diseases endemic to the country of origin or of destination), and with the vaccines requested by certain countries (e.g., the yellow fever vaccination required by the International Health Regulations [59]) [60].

## 2. The Study

Given the need to restore tourism, the possibility that SARS-CoV-2 will be an endemic virus that will last for extended periods in many regions [61], and the requirements for booster COVID-19 vaccines for travel reasons, research is needed to assess the intention to get the COVID-19 vaccine for travel purposes. Furthermore, to ensure travel restoration under the existence of vaccination, travel promotion campaigns and communication efforts must use research-based strategies on sociodemographic and psychosocial variables [13]. We also explored the differences in travel behavior between vaccinated and unvaccinated individuals during a relaxation period (several months after lifting the COVID-19 related restrictions) before wave four in Romania. We addressed the following research questions.

- Research Question 1: How likely are individuals to get the COVID-19 vaccine if that the vaccine would facilitate their travel?
- Research Question 2: What are the predictors of vaccination for travel reasons? We expected sociodemographic characteristics, vaccination status, perceived risk of severity of disease at the destination, conspiracy beliefs, beliefs of safety and efficacy of vaccines, and self-efficacy in controlling the disease to predict the intention to get vaccinated for travel.
- Research Question 3: What are the differences between vaccinated and unvaccinated participants regarding travel intention, avoidance, and cautious travel? We expected that vaccinated individuals intend to travel more and are less avoidant than unvaccinated participants.
- Research Question 4: What are the differences between individuals that are vaccinated against COVID-19 and those who are not regarding travel-related cognitive factors (perceived risk of infection and transmission of infection, perceived severity, self-efficacy beliefs, conspiracy beliefs, and beliefs of safety and efficacy of vaccines) during the pandemic? We predicted that vaccinated participants will perceive greater risk and severity related to COVID-19 and safety of vaccines while they will believe less in conspiracy beliefs.

## 3. Materials and Methods

### 3.1. Construct Measures

A cross-sectional design was used to test the hypotheses. Several scales and items adapted from previous relevant studies were administered to ensure content validity. Two translators translated the English instruments into Romanian independently.

#### 3.1.1. Demographic Information

As demographic variables, respondents were asked to indicate their sex (“male”, “female”), their age (in decade categories), their highest educational level obtained (from ”primary school degree” to “postgraduate”), employee status (“employed”, “not-employed”, “student”, “self-employed”, “not-employed due to COVID-19 crisis”, “re-tired”), whether they already got infected by the virus (“yes”, “no”), and whether they were vaccinated or not.

#### 3.1.2. Vaccination Due to Travel Reasons

To measure the vaccination intent, we used the format of the question proposed by Karlsson et al. but adapted to travel reasons [36]. Participants responded to the question on a 5-point Likert-type scale ranging from 1 (very unlikely) to 5 (very likely) (i.e., “How likely is it you will get a COVID-19 vaccine that is freely available and recommended by authorities if it allows you to travel to the desired destination?”).

#### 3.1.3. Perceived Risk of Infection and of Transmitting the Infection

Participants showed the perceived probability of infection at the travel destination by responding on a 5-point Likert-type scale ranging from 1 (very low) to 5 (very high) to questions indicating the perceived probability of getting infected themselves (“How likely do you believe it is that you will get infected with COVID-19 at the travel destination?”) and transmitting the infection to the loved ones (“How likely do you believe it is that you will transmit the COVID-19 infection to the loved ones at return from your travel?”).

#### 3.1.4. Knowledge of COVID-19 at Travel Destination

A 7-point ordinal scale developed by Georgiou et al., (2020) and adapted to travel destinations for this study was used to measure knowledge about COVID-19 infection. Participants rated their knowledge of COVID-19 infection at travel destination: 1 = Unaware of any COVID-19 in the country of travel destination, 2 = COVID-19 in the country of travel destination, 3 = COVID-19 in the city of travel destination, 4 = COVID-19 in the local area of travel destination, 5 = Persons you know were infected at the travel destination, 6 = Someone close to you was infected at the travel destination, 7 = Currently or have been affected by COVID-19 in travel destination. [62].

#### 3.1.5. Perceived Severity of Infection at Destination

Participants indicated the perceived severity of infection by responding on a 5-point Likert-type scale ranging from 1 (no severity) to 5 (very severe) to the following question: “If you got infected with COVID-19 at the travel destination, how threatening would it be to your physical health?”.

#### 3.1.6. Travel-Related Measures

Intention to travel (three items, e.g., “Whenever I have a chance to travel, I will” [63]), avoidance of travel (two items, e.g., “I will avoid travelling in the post-COVID-19 period” [64]), and cautious travel (three items, e.g., “I try to manage the risk of infection during travel” [64]) were measured. Participants responded to each item on a 5-point Likert-type scale ranging from 1 (strongly disagree) to 5 (strongly agree) (Cronbach’s alpha = 0.85–0.92).

#### 3.1.7. Self-Efficacy for Controlling SARS-CoV-2 Infection

A four-item scale measuring self-efficacy developed by Zheng et al. based on previous work [65,66] and adapted to controlling COVID-19 infection in relation to travel [64] was used in the study. Participants responded to each item (e.g., “I have the necessary skills and equipment to protect myself from being infected by COVID-19”) on a 4-point Likert-type scale ranging from 1 (strongly disagree) to 4 (strongly agree). The internal consistency of the scale was good in our sample (Cronbach’s alpha = 0.72).

#### 3.1.8. Beliefs about Vaccine Safety and Efficacy in Preventing SARS-CoV-2 Infection

A five-item scale measuring beliefs of vaccine efficacy in controlling the COVID-19 infection was developed for the study. Participants responded to each item on a 4-point Likert-type scale ranging from 1 (strongly disagree) to 4 (strongly agree). The first item indicated the perception of safety of vaccine (i.e., ”I believe COVID-19 vaccine is safe”) while the other four indicated whether the person perceives that the vaccine is able to protect them against each major risk related to COVD-19 (“I believe COVID-19 vaccine protects me from being infected with coronavirus if I travel”; “I believe COVID-19 vaccine protects me from developing a severe disease due to Coronavirus infection if I travel”; “I believe COVID-19 vaccine protects me from dying due to Coronavirus infection if I travel”; “I believe COVID-19 vaccine protects me from transmitting COVID to other people if I travel”.) The internal consistency of the scale was excellent in our sample (Cronbach’s alpha = 0.93).

#### 3.1.9. The COVID-19 Conspiracy Beliefs Scale 

This is a short 6-item scale developed by Allington et al. that measures conspiracy beliefs about COVID-19 [67]. Participants were asked to rate whether they believe each statement (e.g., “The current pandemic is part of a global effort to force everyone to be vaccinated whether they want to or not”) is true on a two-point scale, choosing 1 (true) or 0 (false). The internal consistency of the scale was good in the current sample (Cronbach’s alpha = 0.78).

### 3.2. Data Collection

Data regarding vaccination variables from a study on Travel and Personality during COVID-19 pandemic were used for the present study (https://doi.org/10.3390/ijerph182111169, accessed on 10 November 2021). A cross-sectional study was conducted among Romanian residents who experienced the outbreak of COVID-19 within Romania and were interested in traveling. An online form was generated via Google.forms and sent to individuals in Bihor County via Facebook groups, county groups promoting tourism, and by email. Together with the convenience sample method, a snowballing strategy was used asking participants willing to complete the scales to send the form to other people they know.

The study was conducted from 25 May to 1 October 2021, which is approximately one year and two months after quarantine restrictions in Romania and just before the fourth wave in Romania. After giving informed consent, the 110 participants completed the questionnaires. The study was conducted in accordance with the guidelines of the Declaration of Helsinki and approved by the Ethics Committee of the Faculty of Geography, Tourism, and Sport, University of Oradea (approval number 01/2021). Data regarding vaccine intention and factors affecting vaccine intention were analyzed for the present study.

Because our study included individuals who had been already vaccinated, comparing vaccinated vs. unvaccinated people regarding attitudes toward COVID-19 and vaccination helps build effective strategies for increasing the vaccination rates in regions with low vaccination rates, such as Romania and beyond.

### 3.3. Data Analysis

Descriptive analyses of percentage of responses given by vaccinated and unvaccinated respondents were presented. In order to analyze the associations between the variables, parametric and non-parametric correlational analyses were used. The statistical model involved MANOVA tests for comparing the group differences between vaccinated and unvaccinated participants. Two MANOVA tests were carried to analyze the differences between variables. First, a MANOVA test was carried out to compare differences between vaccinated vs. unvaccinated respondents regarding intention to travel, travel avoidance and cautious travel. Second, a MANOVA test was carried out to compare differences between vaccinated vs. unvaccinated respondents regarding perceived risk (for self and others) and perceived severity of infection during travel, conspiracy beliefs, self-efficacy in controlling the COVID-19 infection, and vaccine safety and efficacy. To examine individual mean difference comparisons across analyzed variables a series of post-hoc analyses (Fisher’s LSD) were performed. An alpha threshold of *p* < 0.05 for significance was set for post-hoc analyses. G*Power analyses suggest that a sample of 98 participants assures sufficient power (0.80) to detect small effect sizes (0.15) for linear multiple regression analyses with six predictors (alpha = 0.05, two tailed). Furthermore, 55 participants assure sufficient power to detect a small effect size for simple linear regression analyses. We planned to select 110 participants to ensure sufficient power for the planned analyses. We performed simple regression analyses entering each variable as a predictor and the intention to get vaccinated as the outcome variable. Further, we performed a multiple linear regression, simultaneously entering all previous significant predictors and the intention to get vaccinated due to travel reasons as dependent variable. The regression models were checked for multicollinearity. All values of the variance inflation factor (VIF) were below the maximum threshold level of 10. The analyses were carried out using IBM SPSS Statistics for Windows, Version 23.0, IBM Corp, Armonk, NY, USA.

## 4. Results

### 4.1. Demographics 

Demographics Are Described in Table 1 below.

### 4.2. The Intent to Vaccinate against COVID-19 Due to Travel Reasons (Research Question 1)

When asked how likely they were to get the COVID-19 vaccine if that vaccine would facilitate the travel, 50.9% respondents considered that it was likely for them to vaccinate if the vaccine would facilitate travel (36.4% very likely and 14.5% likely), while for 14.5% it was hard to say. Table 2 presents the percentage of respondents endorsing the intention to vaccinate for travel reasons.

Among the vaccinated respondents, 74% responded that they would vaccinate for travel reasons, suggesting that previously vaccinated individuals are open to further vaccination for travel reasons. Thus, the requirements of vaccination for travel reasons may spur the openness for another booster dose in individuals who have already been vaccinated.

### 4.3. Predictors of COVID-19 Intention to Vaccinate for Travel (Research Question 2)

In bivariate analyses, variables that had positive associations with the intention to vaccinate for travel included age (r = 0.20, *p* = 0.034), vaccine safety and efficacy beliefs (r = 0.70, *p* < 0.001), vaccination status (Spearman’s rho = 0.67, *p* < 0.001), self-efficacy (r = 0.24, *p* = 0.01), and conspiracy beliefs (r = -0.60, *p* < 0.001). Positive correlations were observed between all travel behaviors and the intention for vaccination, including intention for travel (r = 0.23, *p* = 0.013), cautious travel (r = 0.32, *p* = 0.001), and travel avoidance (r = 0.19, *p* = 0.047). Only variables having significant relationships with the vaccination for travel were included in subsequent analyses. See Table 3 for all bivariate analyses.

Multiple linear regression analyses can be found in Table 3.

To investigate which predictors uniquely explained the variation in the intention to vaccinate for travel, all significant dichotomous and continuous predictors (age, vaccination status, vaccine safety and efficacy beliefs, conspiracy beliefs, and intention to travel) were simultaneously entered into a multiple regression model. This model explained 60% of the variance in the intention to vaccinate for travel (F(6, 103) = 26.35, *p* < 0.001). The predictors of age, conspiracy beliefs, self-efficacy, and intention to travel did not significantly predict the intention to vaccinate for travel, whereas vaccination status (β = 1.03, *p* = 0.001) and beliefs about vaccine safety and efficacy (β = 0.13, *p* < 0.001) independently predicted the intention to vaccinate for travel. Table 4 provides the standardized regression coefficients of the predictors in simple regressions and in the multiple regression model.

### 4.4. Travel Intention, Avoidance, and Cautious Travel in Vaccinated vs. Unvaccinated Respondents (Research Question 3)

The MANOVA test showed a statistically significant difference in reported travel intentions based on vaccination status, F(3, 106) = 6.15, *p* < 0.001; Wilk’s Λ = 0.85, partial η2 = 0.14. A series of post-hoc analyses (Fisher’s LSD) was performed to examine individual mean difference comparisons across reported travel intentions. The results revealed that post-hoc mean comparisons for travel intention and travel avoidance were statistically non-significant (*p* > 0.05).

Significant differences have emerged regarding cautious travel (*p* < 0.001). Vaccinated respondents (M = 4.41, SD = 0.51) indicated greater levels of cautious travel than unvaccinated respondents (M = 3.64, SD = 0.74, large effect size, Cohen’s d = 1.91).

### 4.5. Perceived Risk and Severity of Infection during Travel, Vaccine Efficacy, Conspiracy Beliefs, and Self-Efficacy Beliefs in Vaccinated vs. Unvaccinated Respondents (Research Question 4)

The MANOVA test showed that there was a statistically significant difference in attitude based on vaccination status, F(6, 103) = 12.72, *p* < 0.001; Wilk’s Λ = 0.57, partial η2 = 0.42. Table 5 presents the results of the post-hoc analyses (Fisher’s LSD) of individual mean difference comparisons across perceived risk and severity, conspiracy beliefs, self-efficacy, and vaccine efficacy.

That is, on average, unvaccinated respondents have higher levels of conspiracy beliefs and perceive vaccines as less safe and less able to protect them from disease and death than vaccinated participants.

## 5. General Discussion

We examined the intent to vaccinate for travel reasons and how vaccination status influences intention to get vaccinated for travel in a sample of young travel consumer participants. We further examined the factors that need to be addressed after vaccination to further promote travel. We found age, vaccination status, beliefs of safety and efficacy of vaccines, and conspiracy beliefs predicted the intention to get vaccinated for travel reasons. Importantly, the strongest predictor of intentions to vaccinate against COVID-19 infection was the degree to which respondents believed the vaccine to be safe and effective against COVID-19 infection at the travel destination. Thus, young individuals interested in travel who believe that vaccines will keep them safe and effective are more inclined to vaccinate against COVID-19 if the vaccine allows them to travel as they wish. This is in line with previous studies on yellow fever and Malaria [39,40] that found that the vaccine safety predicts vaccination for travel, and with studies investigating factors that predict COVID-19 vaccination [23,36,68]. This means that individuals who consider vaccines to be safe and effective will vaccinate regardless of the perceived risk and conspiracy beliefs. Thus, we enforce the idea that perceived safety and efficacy of vaccines in preventing the COVID-19 infection and its consequences is an important factor for vaccination for travel reasons in young people interested in travel.

Contrary to our hypothesis and some previous findings [39], we did not find that perceived risk and severity of COVID-19 infection was related to the intention to vaccinate for travel reasons. Yet, our results are in line with studies of Karlsson et al. [36] and Faasse and Newby [68] who found that perceptions of risk of COVID-19 were not important to vaccine intention, and with the findings of Du et al. [33] who found that severity of infection is not related to vaccine hesitancy. Although these results show that personal perceived risk and severity of disease at travel destination is not important for intention to vaccinate for travel, it is possible that when high risk is perceived people will avoid travel rather than vaccinate. Nonetheless, the perceived efficacy of vaccines in preventing severe illness and death seems to be more important. Exploratory analyses using partial correlations show that controlling for the travel avoidance has no influence on the relation between perceived risk/severity and vaccination intention, which favors the first interpretation of little importance of personal risk.

We replicated previous findings showing the role of age in vaccination against COVID-19, showing that younger persons are less probable to vaccinate for travel reasons [23,37,38,46].

We also found a significant relationship between conspiracy beliefs and vaccination for travel. Individuals with higher levels of conspiracy beliefs are less likely to vaccinate for travel reasons. This result adds to studies that show that people with higher levels of conspiracy belief have more vaccine hesitancy [23]. Thus, factors that influence vaccination decisions seem to influence vaccination intent for travel as well. We found a significant relationship between intention to travel and vaccination intention. It is possible that individuals who have a more pronounced desire to travel will be more open to vaccinating if the vaccine will allow them to travel as they wish.

We found that only 25% of unvaccinated participants endorsed as likely or very likely they would vaccinate if the vaccine would facilitate travel, 45% responding they most probably would not vaccinate. On the contrary, 82% of previously vaccinated individuals endorsed as likely (8%) or very likely (74%) that they would vaccinate if the vaccine would facilitate travel. This result adds to studies showing that previous vaccination or vaccination refusal is an important predictor of the intention to vaccinate [3,23,37,38]. Similar results have been found regarding the acceptability of vaccination for domestic or international travel certificates. de Figueiredo et al. found that 78.71% (74% our study) of adult UK participants who received a first dose of vaccine will ‘definitely’ accept a future dose for a domestic or international travel certificate, while a further 11.7% (8% our study) are ‘unsure but leaning towards yes’ [69]. Furthermore, 4.5% (6% in our study) say they will ‘definitely not’ accept a future COVID-19 vaccine, while 5% (4% in our study) are ‘unsure but leaning towards no’. Willingness to vaccinate for travel is also in line with surveys on Romanian population that suggest that traveling is an important reason for why individuals accept vaccines [24].

Contrary to our prediction, we did not find that vaccinated individuals have higher intention to travel and less avoidance of travel. Yet, we found that vaccinated individuals travel more cautiously. We may interpret these findings based on the findings that unvaccinated people have higher levels of conspiracy beliefs and due to not believing in COVID-19 infection probably have similar levels of travel as before the COVID-19 pandemic. It is possible that a cognitive profile of being less cautious, perceiving less risk and severity of disease, and higher conspiracy beliefs may be characteristic of participants who are not vaccinated and do not intend to vaccinate.

Our results suggest that individuals with higher levels of conspiracy beliefs behave differently in response to COVID-19 infection than individuals with low levels of conspiracy beliefs regarding travel intention and travel avoidance.

We found that unvaccinated respondents have higher levels of conspiracy beliefs, perceive vaccines as less safe and less able to protect them from disease and death than vaccinated participants. Thus, we observed higher levels of conspiracy theories and less trust in the safety of the vaccine and its ability to protect individuals against disease and death. It is possible that these large differences in individual characteristics add to social (low trust in institutions) and campaign (messages that limit freedom and impose vaccination) factors resulting in vaccination refusal in the Romanian population. Although other factors are most probably involved in vaccine refusal, previous studies showed that conspiracy beliefs [23] and low perceptions of safety of vaccines [36] are major contributors to vaccine refusal. Consistently with our results, national representative surveys in Romanian population identified as main reasons for vaccine refusal potential side effects of vaccines (48%), vaccines not being sufficiently tested (54%), or not believing in the effectiveness of vaccines (41%) [24].

Our analyses identify beliefs about the efficacy of vaccines as the best predictors of the intention to vaccinate for travel. These results have several implications for current campaigns and communication strategies. First, travel reasons are good reasons for vaccination campaigns. Focusing on travel benefits after vaccination would stimulate the rate of vaccination. Low rates of vaccination at local tourism destinations [70,71,72,73] may be important obstacles for both foreign travelers who avoid places where only a few people are vaccinated and to travelers who may not travel in safe conditions.

Second, our results suggest that campaigns that promote vaccination for travel only are not enough. The campaigns should simultaneously supply messages that increase trust in the safety of vaccines and the efficacy of vaccination in protection against COVID-19 infection. Presentation movies and flyers about how being vaccinated may provide safety at travel destinations may be a source for vaccine-efficacy beliefs. Less important may be the personal risk perceived by individuals. Thus, messages centered on the accentuation of risk and severity may have little efficacy in increasing the rate of vaccination. On the contrary, messages about the safety and efficacy of vaccines may have a higher impact on intention to vaccinate. Third, conspiracy beliefs are an important predictor of willingness to vaccinate. Campaigns for the promotion of vaccination should include adequate information and systematic countering of fake messages that fuel conspiracy beliefs. This finding underlines previous recommendations for promoting vaccination based on enhancing knowledge about vaccines. Gallè et al. found knowledge about vaccines and prior influenza vaccination to be the main predictors of acceptance of vaccination in an Italian undergraduate sample [3]. Thus, campaigns aiming at increasing proper knowledge about vaccines may be of utility in an undergraduate population. It is possible that a personalized strategy would have an advantage when designing vaccination campaigns. In some samples such as undergraduate participants, general knowledge will be a better target for the campaign, whereas in other samples such as young travel consumers, vaccine efficacy beliefs should be the targeted.

Our current findings, however, should be interpreted considering several limitations. First, exclusive reliance on self-report questionnaires limited them. It would be beneficial for future research to incorporate observation measures. The limited number of participants imposed limitations on interpreting the results. Additional studies should adopt larger samples to confirm our findings. The recruitment of participants occurred via social media tourist groups. Thus, the sample is skewed toward younger respondents. Moreover, having more women than men in our sample limits the generalizability of our findings. Women are more risk aversive and more avoidant than men. Although our sample had a slightly higher number of vaccinated participants (45.5%) than the Romanian population, this percentage is closed to the number of vaccinated individuals officially reported in the city of Oradea where our study took place (40.42%) [60]. Another limit is the self-selection bias. We disseminated the survey via Facebook groups of the county, student groups, and by email. It is possible that travel behavior in these individuals differs from the general population. In addition, we investigated residents’ travel behavior based on travel intentions and self-reported travel. It is possible that there are differences between self-reports and actual travel reporting influenced by factors at the moment of completion of the survey. Further studies should compare self-report and objective data together.

## 6. Conclusions

In conclusion, we found age, vaccination status, conspiracy beliefs, and the perceived safety and efficacy of vaccines to predict the intention to vaccinate due to travel reasons. In the future, considering the endemic nature of COVID-19, measures that target these factors may mitigate vaccination for travel reasons. Significant differences found in this study may be important to describe differences between vaccinated and unvaccinated individuals in Romania before wave four of COVID-19. It seems that the main difference was higher levels of conspiracy beliefs and lower levels of perceptions of vaccine safety and efficacy in unvaccinated individuals. Targeting fake news and adequate vaccination campaigns focused on the safety and effectiveness of vaccines should be priorities to promote vaccination.

## Figures and Tables

**Table 1:** Demographics and Main Variables.

Variable		Frequency	%
Age (years)	0–18	-	-
	19–30	51	46.4%
	31–45	47	42.7%
	45–60	11	10.0%
	60+	-	-
Gender (F/M, *n*, %)	Male	25	31.8%
	Female	75	68.2%
Education (years of study)	High-school	27	24.5%
	Undergraduate	41	37.3%
	Postgraduate	41	37.3%
Income	<250 EUR	34	30.9%
	250–450 EUR	17	15.5%
	450–900 EUR	34	30.9%
	>900 EUR	25	22.7%
Employment	Employed	64	52.8%
	Not employed	4	3.6%
	Not employed due to pandemic	3	2.7%
	Student	33	30.0%
	Retired	1	0.9%
	Self-employed	5	4.5%
Infection and vaccination status	Being infected with coronavirus	23	20.9%
	Having family, neighbors or close friends infected with coronavirus	86	78.2%
	Vaccinated against SARS-CoV-2 or programmed for vaccination	50	45.5%
Knowledge of COVID-19 at travel destination	I don’t know about the existence of COVID-19 in the destination country	15	13.6%
	COVID-19 is present in the destination country	9	44.5%
	COVID-19 is present in the destination city	19	17.3%
	COVID-19 is present in the destination area	22	20.0%
	Persons you know have been infected in the destination you visit	2	1.8%
	Close persons have been infected in the destination you visit	3	2.7%
Perceived risk for self	High and very high	6	5.4%
	Medium	46	41.8%
	Low and very low	58	52.7%
Perceived risk for others	High and very high	30	27.3%
	Medium	33	30.0%
	Low and very low	47	42.8%
Perceived severity for self	High and very high	16	14.6%
	Medium	31	28.2%
	Low and very low	63	57.3%
		Mean	SD
	Conspiracy beliefs	1.93	1.83
	Intention to travel	4.09	0.95
	Self-efficacy	2.04	0.63
	Vaccine safety and efficacy beliefs	12.08	4.87

**Table 2:** Percentage of responses regarding the intention to vaccinate for travel “How likely is it you will get a COVID-19 vaccine that is freely available and recommended by authorities, if it allows you to travel to the desired destination?”.

Vaccination Status	Very Likely	Likely	Hard to Say	Unlikely	Very Unlikely
Vaccinated	74%	8%	8%	4%	6%
Unvaccinated	5%	20%	20%	10%	45%
Total	36.4%	14.5%	14.5%	7.3%	27.3%

**Table 3:** Correlation Coefficients between Vaccination Intention and Perceived Exposure, Risk, Self-Efficacy, Vaccine Safety, Vaccination Status, Conspiracy Beliefs and Intention to Travel.

Variables	1	2	3	4	5	6	7	8	9
1. Vaccination intention	1	-	-	-	-	-	-	-	-
2. Perceived risk for self	0.08	1	-	-	-	-	-	-	-
3.Perceived risk for others	−0.03	0.53 *	1	-	-	-	-	-	-
4. Perceived severity for self	0.01	0.43 *	0.57 **	1	-	-	-	-	-
5. Self-efficacy	0.24 *	0.11	−0.13	−0.10	1	-	-	-	-
6. Vaccine safety and efficacy beliefs	0.70 **	0.18	0.05	0.13	0.22 *	1	-	-	-
7. Prior vaccination	0.67 **	0.02	0.00	0.03	−0.14	−0.59 **	1	-	-
8. Conspiracy beliefs	−0.60 **	−0.10	0.00	0.03	0.14 **	0.61 **	−0.56 **	1	-
9. Intention to Travel	0.23 *	0.06	−0.09	−0.15	−0.14	−0.15 **	0.09 **	−0.20 *	1
10. Cautious travel	0.32 *	0.42 **	0.25 *	0.35 **	−0.32 **	−0.41 **	0.37 *	−0.34 **	0.07

* *p* < 0.05 (2-tailed); ** *p* < 0.001 (2-tailed).

**Table 4:** Regression Analysis of Predictors of the Intention to Vaccinate against COVID-19 for Travel.

	Predictors	β	t	*p*	CI	Part Corr.
Linear Simple Regression Analyses for Main Predictors	Age	0.46	2.08	0.03	[0.02; 0.91]	0.19
	Gender	0.25	0.75	0.45	[−0.41; 0.92]	0.07
	Perceived risk of infection for self	0.16	0.90	0.36	[−0.19; 0.51]	0.08
	Perceived risk of transmitting the infection to others	−0.05	−0.37	0.70	[−0.32; 0.21]	−0.03
	Perceived severity of infection at TD	0.01	0.10	0.91	[−0.30; 0.34]	0.01
	Knowledge of COVID-19 at TD	−0.02	−0.18	0.85	[−0.29; 0.24]	−0.01
	Vaccination status	2.10	8.57	0.00	[1.61; 2.58]	0.63
	Conspiracy beliefs	−0.54	−7.87	0.01	[−0.68; −0.40]	−0.60
	Vaccine safety and efficacy beliefs	0.23	10.25	0.00	[0.19; 0.28]	0.70
	Self-Efficacy	0.63	2.57	0.01	[0.14; 1.11]	0.24
	Intention to travel	0.40	2.53	0.13	[0.08; 0.72]	0.23
Linear Multiple Regression Analysis Equation	Age	−0.12	−0.74	0.45	[−0.45; 0.20]	−0.04
	Vaccination status	1.03	3.57	0.01	[0.45; 1.60]	0.19
	Conspiracy beliefs	0.13	1.83	.069	[−0.29; 0.01]	−0.11
	Vaccine safety and efficacy beliefs	0.13	4.70	0.00	[0.07; 0.19]	0.29
	Self-Efficacy	0.16	0.98	0.32	[−0.50; 0.16]	0.06
	Intention to Travel	0.17	1.58	0.11	[−0.04; 0.39]	0.09

TD—travel destination; Part Corr.—Part correlations.

**Table 5:** Percentage of responses regarding the intention to vaccinate for travel.

Variable	Perceived Risk of Infection for Self	Perceived Risk of Transmitting the Infection to Others	Perceived Severity of Infection	Self-Efficacy of Controlling COVID-19 Infection	Vaccine Safety and Efficacy Beliefs	Conspiracy Beliefs
Significance of the mean difference	*p* > 0.05 Cohen’s d = 0.02	*p* > 0.05 Cohen’s d = −0.01	*p* > 0.05 Cohen’s d = 0.06	*p* > 0.05 Cohen’s d = 0.29	*p* < 0.001 Cohen’s d = 1.45	*p* < 0.001 Cohen’s d = 1.37

## Data Availability

Available on request from the corresponding author.
